# Shared genetic loci between Alzheimer’s disease and multiple sclerosis: Crossroads between neurodegeneration and immune system

**DOI:** 10.1016/j.nbd.2023.106174

**Published:** 2023-06-05

**Authors:** Vera Fominykh, Alexey A. Shadrin, Piotr P. Jaholkowski, Shahram Bahrami, Lavinia Athanasiu, Douglas P. Wightman, Emil Uffelmann, Danielle Posthuma, Geir Selbæk, Anders M. Dale, Srdjan Djurovic, Oleksandr Frei, Ole A. Andreassen

**Affiliations:** a NORMENT Centre, Institute of Clinical Medicine, University of Oslo, Oslo, Norway; b Division of Mental Health and Addiction, Oslo University Hospital, Oslo, Norway; c Department of Complex Trait Genetics, Centre for Neurogenomics and Cognitive Research, Amsterdam Neuroscience, Vrije Universiteit Amsterdam, Amsterdam, the Netherlands; d Department of Child and Adolescent Psychiatry and Pediatric Psychology, Section Complex Trait Genetics, Amsterdam Neuroscience, Vrije Universiteit Medical Center, Amsterdam, the Netherlands; e Department of Geriatric Medicine, Oslo University Hospital, Oslo, Norway; f Norwegian National Centre for Ageing and Health, Vestfold Hospital Trust, Tonsberg, Vestfold, Norway; g Department of Radiology, University of California San Diego, La Jolla, California, USA; h Multimodal Imaging Laboratory, University of California San Diego, La Jolla, California, USA; i Department of Psychiatry, University of California San Diego, La Jolla, California, USA; j Department of Neurosciences, University of California San Diego, La Jolla, California, USA; k Department of Medical Genetics, Oslo University Hospital, Oslo, Norway; l K.G. Jebsen Centre for Neurodevelopmental disorders, University of Oslo and Oslo University Hospital, Oslo, Norway; m Department of Informatics, Centre for Bioinformatics, University of Oslo, Norway

**Keywords:** Alzheimer’s disease, Multiple sclerosis, Genetic overlap, Pleiotropy, Dementia, Neurodegeneration, Neuroinflammation

## Abstract

**Background:**

Neuroinflammation is involved in the pathophysiology of Alzheimer’s disease (AD), including immune-linked genetic variants and molecular pathways, microglia and astrocytes. Multiple Sclerosis (MS) is a chronic, immune-mediated disease with genetic and environmental risk factors and neuropathological features. There are clinical and pathobiological similarities between AD and MS. Here, we investigated shared genetic susceptibility between AD and MS to identify putative pathological mechanisms shared between neurodegeneration and the immune system.

**Methods:**

We analysed GWAS data for late-onset AD (N cases = 64,549, N controls = 634,442) and MS (N cases = 14,802, N controls = 26,703). Gaussian causal mixture modelling (MiXeR) was applied to characterise the genetic architecture and overlap between AD and MS. Local genetic correlation was investigated with Local Analysis of [co]Variant Association (LAVA). The conjunctional false discovery rate (conjFDR) framework was used to identify the specific shared genetic loci, for which functional annotation was conducted with FUMA and Open Targets.

**Results:**

MiXeR analysis showed comparable polygenicities for AD and MS (approximately 1800 trait-influencing variants) and genetic overlap with 20% of shared trait-influencing variants despite negligible genetic correlation (rg = 0.03), suggesting mixed directions of genetic effects across shared variants. conjFDR analysis identified 16 shared genetic loci, with 8 having concordant direction of effects in AD and MS. Annotated genes in shared loci were enriched in molecular signalling pathways involved in inflammation and the structural organisation of neurons.

**Conclusions:**

Despite low global genetic correlation, the current results provide evidence for polygenic overlap between AD and MS. The shared loci between AD and MS were enriched in pathways involved in inflammation and neurodegeneration, highlighting new opportunities for future investigation.

## 1. Introduction

Alzheimer’s disease (AD) is the most common forms of dementia, characterised by progressive neurodegeneration and specific neuropathological features (Jack Jr et al., 2016). AD is a major health problem in most countries, with high costs for health services and society (Abdelnour et al., 2022). Usually, the clinical features of AD include specific memory decline, apathy, anxiety, and depression in earlier stages, with conversion to impaired communication, psychotic symptoms, confusion, and disorientation at the late stages (Jack Jr et al., 2016). The pathophysiology of AD is characterised by the accumulation of amyloid-beta (Aβ) and hyperphosphorylated tau protein, which lead to the production of amyloid plaques and neurofibrillary tangles (Golde, 2022). After the *TREM2* gene discovery (Jonsson et al., 2013; Sherva et al., 2022), research in immunological mechanisms implicated in AD has increased (Piller, 2022). Chronic activation of the immune cells in the brain and immune cell trafficking from the periphery via the blood-brain barrier contributes to the neuroinflammatory response, which is involved in the neurodegenerative processes in AD (Leng and Edison, 2021). Further, immune-based drugs preventing Aβ accumulation have shown promising effects in early AD (Van Dyck et al., 2023; Thambisetty and Howard, 2023).

Most AD cases emerge after 65 years of age (late-onset AD) and have complex genetic inheritance without a clear family history. Estimated heritability for AD from twin studies is 58–79% (Gatz et al., 2006), and estimated SNP heritability from genome-wide association studies (GWAS) is 3.1–6.9% (Lambert et al., 2013; Wightman et al., 2021; Bellenguez et al., 2022). A small number (<1%) of AD cases are familial, with mainly early onset (Tanzi, 2012), and associated with rare, genetic mutations in amyloid-pathway genes (Campion et al., 1999). Other early-onset cases are mostly associated with the same rare/common variants as late-onset AD (eg, *TREM2*, *SORL1*, *RUFY1*, *PSD2*, *RIN3*, and others, Kunkle et al., 2017). For late-onset AD, the genetics is more complex. The most well-known genetic risk factor is apolipoprotein E (*APOE*), yet there is also a strong polygenic component. Moreover, rare mutations, for example, the mutation in microtubule-associated protein tau gene (*MAPT*, Strang et al., 2019) and common variants (Wightman et al., 2021), contributed to late-onset AD. The most recent late-onset AD GWAS identified 75 AD risk loci (Bellenguez et al., 2022). Functional annotation of these loci revealed the involvement of microglia, immune cells, and protein catabolism, which can be relevant for developing new treatment regimens. Several implicated genes, such as *TNIP1*, *LILRA5*, *TREM1*, *HLA-DRB1*, *CR1*, *GLU*, play an essential role in the function of the immune system. Furthermore, proteomic studies in AD showed the involvement of molecular pathways linked to neuroinflammation, such as proinflammatory interleukins and cytokines molecules (Wingo et al., 2021).

Multiple sclerosis (MS) is a chronic, immune-mediated, and neurodegenerative disease of the central nervous system (Waubant et al., 2019). MS has a strong genetic influence, and large GWAS have identified 200 non-major histocompatibility complex (MHC) and 32 MHC-loci associated with MS, with a SNP heritability (h2) around 48% (Patsopoulos et al., 2019). Immune-mediated myelin damage is important in disease activity and progression, and neuroinflammation is the key component of the pathogenesis of MS. Further, anti-inflammatory and immunomodulatory treatments are effective in relapsing-remitting MS and work at the earlier stages of the disease (Yang et al., 2022). However, these medications have a small impact in progressive MS and late stage of the disease due to the activation of neurodegenerative processes, which start at early stages of MS (Kaufmann et al., 2022) and suggest partly overlapping disease mechanisms with neurodegenerative diseases such as AD (Amezcua, 2022). Further, shared peripheral immunity mechanism was shown between AD and MS (Rossi et al., 2021). Moreover, there is a higher frequency of AD-related dementia (Mahmoudi et al., 2022), and neuropathologically confirmed neurodegenerative pathology in MS (Londoño et al., 2022).

Despite different clinical manifestations of AD and MS, shared genetic risk factors have been previously reported. For example, *APOE4*, the most important genetic risk factor for AD, has been reported to be a predictor of MS velocity, severity, and cognitive decline (Naseri et al., 2022), although there are contradictory results (Masterman et al., 2002). Further, the MHC region and immune-associated genes have been implicated in both diseases. MS is twice as common in women as in men, probably caused by hormonal and genetic factors (Harbo et al., 2013). AD is also more prevalent in women, and this sex difference is related to higher life expectancy in women (Mielke et al., 2014; Beam et al., 2018), genetic architecture, and neurobiological vulnerability in postmenopausal females (Podcasy and Epperson, 2016). There are few cases of comorbid AD and MS (Luczynski et al., 2019) but MS show signs of amyloid pathology in the brain and impaired Aβ metabolism in cerebro-spinal fluid (Petitfour et al., 2022; Johansson et al., 2022; Pietroboni et al., 2017), suggesting overlapping pathobiology.

There is an enormous interest in drug development in AD (Cacabelos, 2022). Many pharmacological companies have focused on developing disease-modifying therapies using different monoclonal antibodies to prevent plaque formation (aducanumab, gantenerumab, lecanemab) and anti-tau therapy with designed tau-neutralised antibodies (Ossenkoppele et al., 2022). While the FDA has approved aducanumab, and lecanemab, the progress in AD drug development is limited due to the small effect of the medication on clinical progression and relation to amyloid build-up (Cummingd et al., 2022). Evidence suggests that drugs are twice as likely to be approved for clinical use if they are supported by GWAS evidence (Nelson et al., 2015). As such, GWAS can be a cost-efficient approach to prioritise new drug targets. Moreover, integrating overlapping genetic associations between AD and MS with multiomics and clinical data may guide the design of immune-related drugs for AD.

Previous reports show shared genetics risk factors between neurodegenerative disorders (AD, Parkinson’s disease, and multiple system atrophy) and different somatic immune-mediated disorders (Shadrin et al., 2021; Witoelar et al., 2017). Additionally, positive associations with both maternal and clinical AD have been reported to be a liability to MS using a multivariable Mendelian randomization study (Yeung et al., 2022). There are few cross-trait GWAS results for MS due to low power in previous GWAS (Andreassen et al., 2014; Elvsåshagen et al., 2020). However, the recent large GWAS from the IMSGC is more powerful (Patsopoulos et al., 2019).

Traditional methods for investigating genetic overlap between diseases include genetic correlation and polygenic risk score, which build on the assumption that genetic effect directions between two traits are predominantly concordant across the genome. This seems not to be the case with brain-related traits where there is a mixture of concordant and discordant directions of genetic effects across overlapping variants (Bahrami et al., 2022; Frei et al., 2019). In contrast, MiXeR (Frei et al., 2019) estimates the total number of trait-specific and shared genetic variants influencing two traits regardless of effect direction, which seems more relevant for investigating molecular pathways of brain-related diseases. Further, conditional-conjunctional false discovery rate (cFDR) method can be used to identify shared genetic loci between traits (Smeland et al., 2020) and Local Analysis of [co]Variant Association (LAVA) for local genetic correlation (Werme et al., 2022).

The present study aims to reveal molecular mechanisms involving the immune system in AD, by investigating shared genetic architecture and overlapping genetic loci between AD and MS. This can pave the way for precision therapeutic strategies targeting neuroinflammation in neurodegenerative processes involved in AD.

## 2. Materials and methods

### 2.1. Samples

**Fig. 1.**
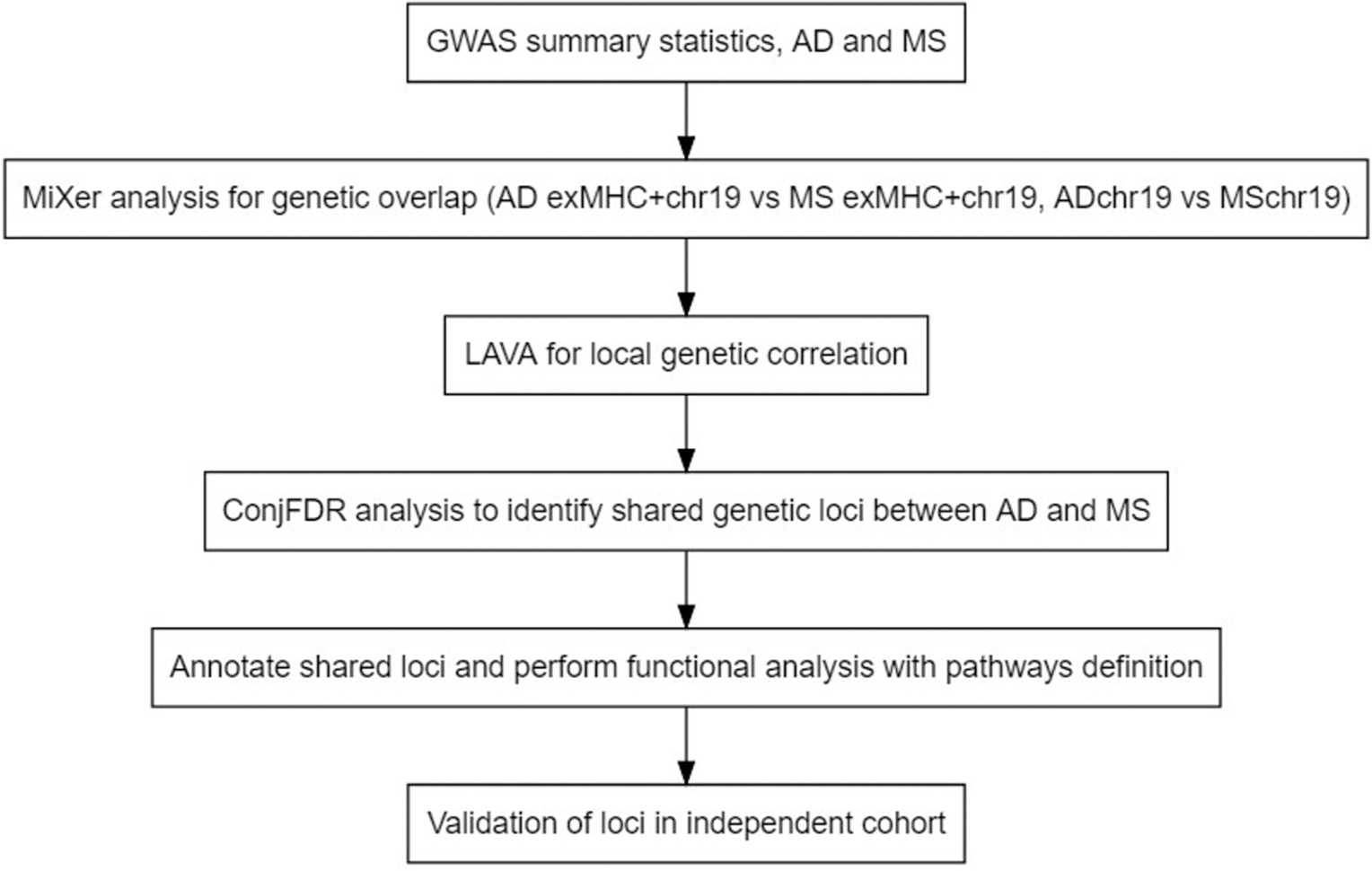
Flowchart of the study design. AD – Alzheimer’s disease, GWAS – genome-wide association study, MS – multiple sclerosis.

GWAS summary statistics for AD from the Psychiatric Genomics Consortium (PGC) were used in this study (Wightman et al., 2021), excluding the 23andMe sample and data from the IGAP consortium due to overlap with control cohorts in the MS GWAS. The summary statistics for AD were partly based on the UKB cohort which included both cases and proxy-cases, and controls (Wightman et al., 2021). The final sample size was 64,549 cases and 634,442 controls. The MS GWAS summary statistics were obtained from the International Multiple Sclerosis Genetics Consortium (IMSGC; discovery phase, 14,802 cases and 26,703 controls, Patsopoulos et al., 2019). Participants in both GWAS were predominantly of European ancestry. Detailed descriptions of cohorts and samples are available in original publications (Wightman et al., 2021, Patsopoulos et al., 2019). For the validation of findings in AD we used summary statistics from an independent dataset with 2784 AD cases and 5222 controls recruited from several case-control and family-based studies of African Americans (Kunkle et al., 2021). A detailed description of the cohorts included in this analysis are provided in the Supplementary Material (Kunkle et al., 2021, Supplementary Note; Supplementary Tables 1-3). A study flowchart is presented in Fig. 1.

### 2.2. Analytical tools

#### 2.2.1. Gaussian causal mixture modelling method (MiXeR)

For investigation of the genetic architecture of AD and MS we used MiXeR software (Frei et al., 2019, https://github.com/precimed/mixer, Shadrin et al., 2020). Due to the high impact of the *APOE* region in AD we followed the analysis setup used in Holland et al. (2020) treating chromosome 19 separately from the other chromosomes. Additionally, we excluded the MHC region (Frei et al., 2019), due to the intricate linkage disequilibrium (LD) structure. Results are presented as Venn diagrams displaying the proportion of trait-specific and shared trait-influencing SNPs followed by the standard deviation across 20 independent runs, log-likelihood plots and tables with parameters estimated by the MiXeR model, explaining 100% of the heritability.

#### 2.2.2. Linkage disequilibrium score regression (LDSC) and local analysis of [co]variant association (LAVA)

For establishing genome-wide genetic correlation (rg) we used LDSC (Bulik-Sullivan et al., 2015) and for local rg analysis we used LAVA (Werme et al., 2022). For LAVA analysis we followed the protocol described in original article using the LD reference panel based on 1000 Genomes phase 3 genotype data for European samples (Werme et al., 2022; 1000 Genomes Project Consortium et al., 2015), and the partition of the genome into 2495 regions with average size of 1 Mb. Only regions revealing significant estimated SNP heritability (*p* < 0.05/2495) in both AD and MS were used to estimate local genetic correlations between the traits.

#### 2.2.3. Quantile-quantile (QQ) plots and conditional false discovery rate (cFDR) analyses

We used conditional quantile-quantile (QQ) plots to visualise polygenic enrichment. Each conditional QQ plot shows the distribution of *P* values in the GWAS of the primary phenotype for a subset of variants selected based on the significance of their association with the conditional phenotype at three levels: *P_secondary* < 0.1, *P_secondary* < 0.01, and *P_secondary* < 0.001. For QQ plots production, we excluded variants within 3 regions: with complex LD patterns (MHC region: chr6:25119106–33,854,733) and regions linked to dementia phenotype (MAPT region: chr17:40000000–47,000,000; *APOE* region: chr19:42000000 47,000,000). Conditional QQ plots were produced in both directions: conditioning AD on MS, and conditioning MS on AD. Successive leftward deflection of the variant strata with increasing significance in the conditional phenotype in both directions suggests genetic overlap between traits. We used cFDR method including conditional FDR (condFDR) and conjunctional FDR (conjFDR) analyses (https://github.com/precimed/pleiofdr, Frei et al., 2019, Andreassen et al., 2013, 2014). We applied condFDR as the first part of the analysis (Supplementary1) and conjFDR analysis to identify shared genetic loci. According to standard protocols, the FDR significance cut-offs were 0.01 for condFDR and 0.05 for conjFDR (Cheng et al., 2021; Smeland et al., 2020).

#### 2.2.4. Validation phase

We examined the significance of the identified lead variants shared between AD and MS in the independent cohort of AD (Kunkle et al., 2021). The sign-concordance test was not performed due to the absence of information about the direction of effect in the validation summary statistics.

#### 2.2.5. Functional annotation of loci and novelty

We followed FUMA protocol for the identification of genomic loci and lead variants (Watanabe et al., 2017) using significance thresholds of condFDR<0.01 and conjFDR<0.05. For novelty checking, we compared our loci with the original GWAS (Wightman et al., 2021, Patsopoulos et al., 2019), National Human Genome Research Institute-–European Bioinformatics Institute GWAS Catalog (GWAS Catalog, ebi.ac.uk), other GWAS for AD and MS (Kunkle et al., 2017, Lambert et al., 2013, Jansen et al., 2019, Kunkle et al., 2021, Schwartzentruber et al., 2021, Marioni et al., 2018, Bellenguez et al., 2022, Escott-Price et al., 2014, Mez et al., 2017, Zhou et al., 2018, Jun et al., 2017), and previous cFDR studies (Drange et al., 2019; Wang et al., 2017; Adewuyi et al., 2022; Broce et al., 2019; Ahangari et al., 2022). Additionally, we checked our results against the ADVP database for AD (Kuksa et al., 2022 https://advp.niagads.org/variants). If the locus was not presented in these sources, it was considered as novel.

We used FUMA Combined Annotation Dependent Depletion (CADD) Score ≥ 12.37 to annotate the most deleterious variants (Rentzsch et al., 2019; Kircher et al., 2014), Regulome Database scores (Boyle et al., 2012) and chromatin states (Kundaje et al., 2015) were used for functional annotation of SNPs with conjFDR<0.05.

We applied two SNP-to-gene mapping strategies, including (1) positional mapping according to FUMA with 10 kb window size and (2) Open Targets modelling for the annotation, which combined positional mapping as a distance between the variant and each gene’s canonical transcription start site, eQTL, pQTL, splicingQTL and epigenomic data, and functional prediction (Ghoussaini et al., 2021, Mountjoy et al., 2021, database version from October 2022). After excluding mapped genes in the MHC region, we applied genes to function analysis as implemented in FUMA. We used the Molecular Signatures Database to evaluate enrichment in immunological gene sets. We also applied MAGMA to summary statistics on AD and MS to test for enrichment of GWAS signals in 54 tissues (de Leeuw et al., 2015). The 54 gene-sets were defined by gene-expression levels from 54 GTEx tissues (GTEx Consortium, 2015).

We used Cytoscape (version 3.9.1, Shannon et al., 2003) with STRING database for pathway analysis.

## 3. Results

### 3.1. Univariate and bivariate MiXeR show similar polygenicity between AD and MS and large genetic overlap despite negligible global genetic correlation

Our LDSC analysis of current GWAS data shows weak non-significant genetic correlation between AD and MS (rg = 0.03, standard error (SE) = 0.07, *P* = 0.67). AD heritability estimated by LDSC was 0.0421, SE = 0.007. MS heritability estimated by LDSC was 0.3059, SE = 0.0256. Univariate MiXeR estimated the SNP heritability (h2) = to 0.088 for AD (h2 for chromosome 19 + h2 for all other autosomes) and 0.249 for MS (h2 for chromosome 19 + h2 for all other autosomes).

**Fig. 2.**
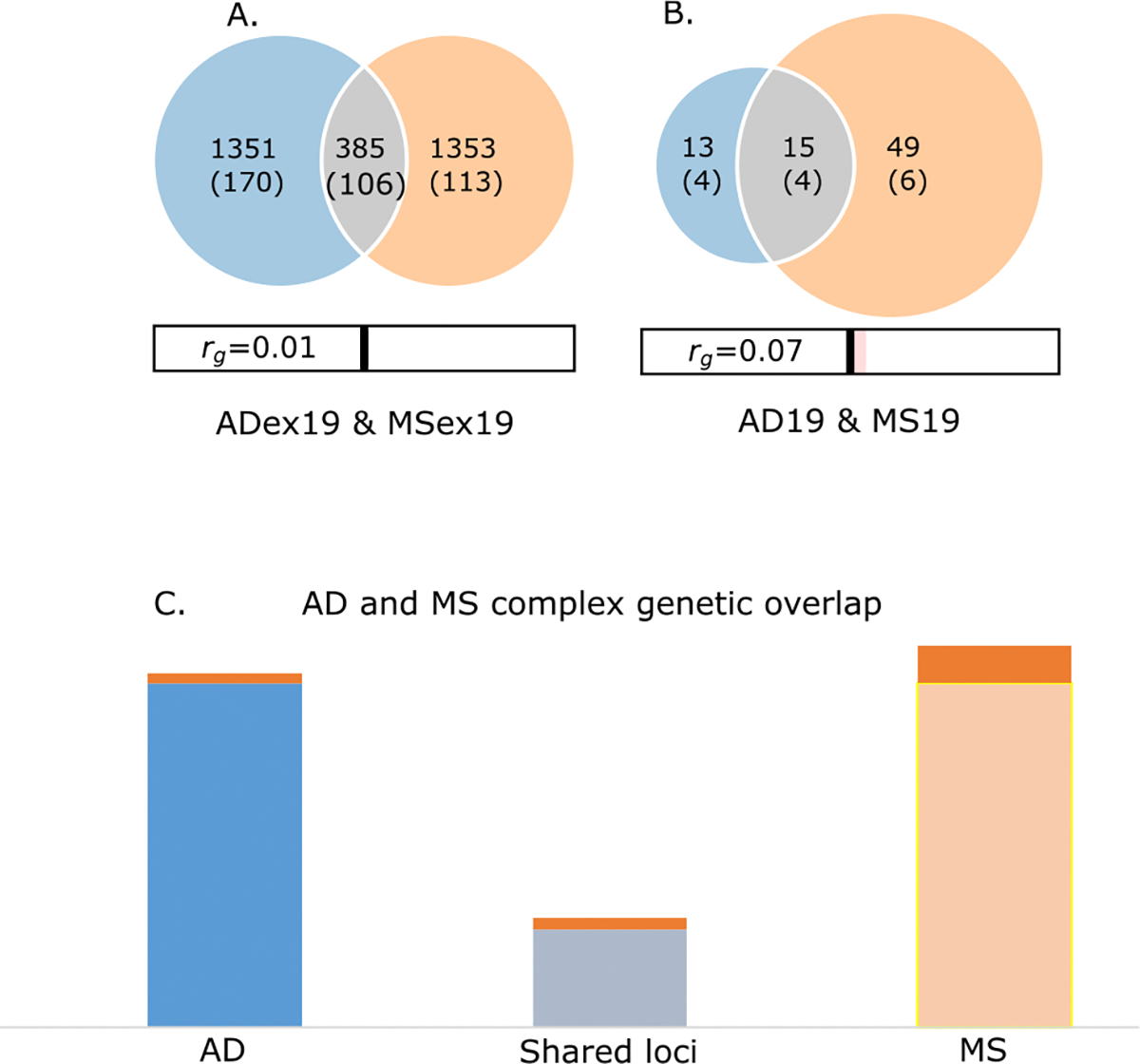
Bivariate MiXeR results: **A:** polygenic overlap between Alzheimer’s disease (AD) and multiple sclerosis (MS). Venn diagram showing the estimated number of causal variants shared between AD and MS in the current settings (grey), and unique to each disorder (AD blue circle, MS orange circle). The size of the circles represents the level of polygenicity for AD and MS (both without chromosome 19 and MHC). The numbers indicate the estimated number of causal variants, followed by the standard deviation across 20 independent runs. **B:** polygenic overlap between AD and MS (chromosome 19). Venn diagram showing the estimated number of causal variants shared between AD and MS on chromosome 19 (grey), and unique to each disorder (AD blue circle, MS orange circle). The size of the circles represents the level of polygenicity for chromosome 19 AD and MS. The numbers indicate the estimated number of causal variants, followed by the standard deviation across 20 independent runs. **C.** MS and AD complex genetic overlap. **Column 1:** AD loci: orange band – loci on chromosome 19, blue band – all other loci. **Column 2**: Shared loci: orange band – shared loci on chromosome 19, grey band – all other loci. **Column 3:** MS loci: dark orange band – loci on chromosome 19, light orange band – all other loci.

Using univariate MiXeR we show that AD and MS show similar polygenicity. The number of trait-influencing variants was estimated to 1763 variants for AD and 1802 for MS. Bivariate MiXeR analysis revealed polygenic overlap on autosomes excluding chromosome 19 (Fig. 2A, and Fig. 1, Supplementary) and for chromosome 19 (Fig. 2B, and Fig, 1, Supplementary) with adequate quality of model fit suggested by log-likelihood plots and Akaike information criteria. For all chromosomes the predicted number of shared loci between MS and AD was around 400, for chromosome 19 specifically it was 15 variants (Fig. 2C). The observed substantial genetic overlap with minor genetic correlation suggests a balanced mixture of concordant and discordant genetic effects across shared loci.

### 3.2. LAVA analysis provides insight into local genetic correlation

**Fig. 3.**
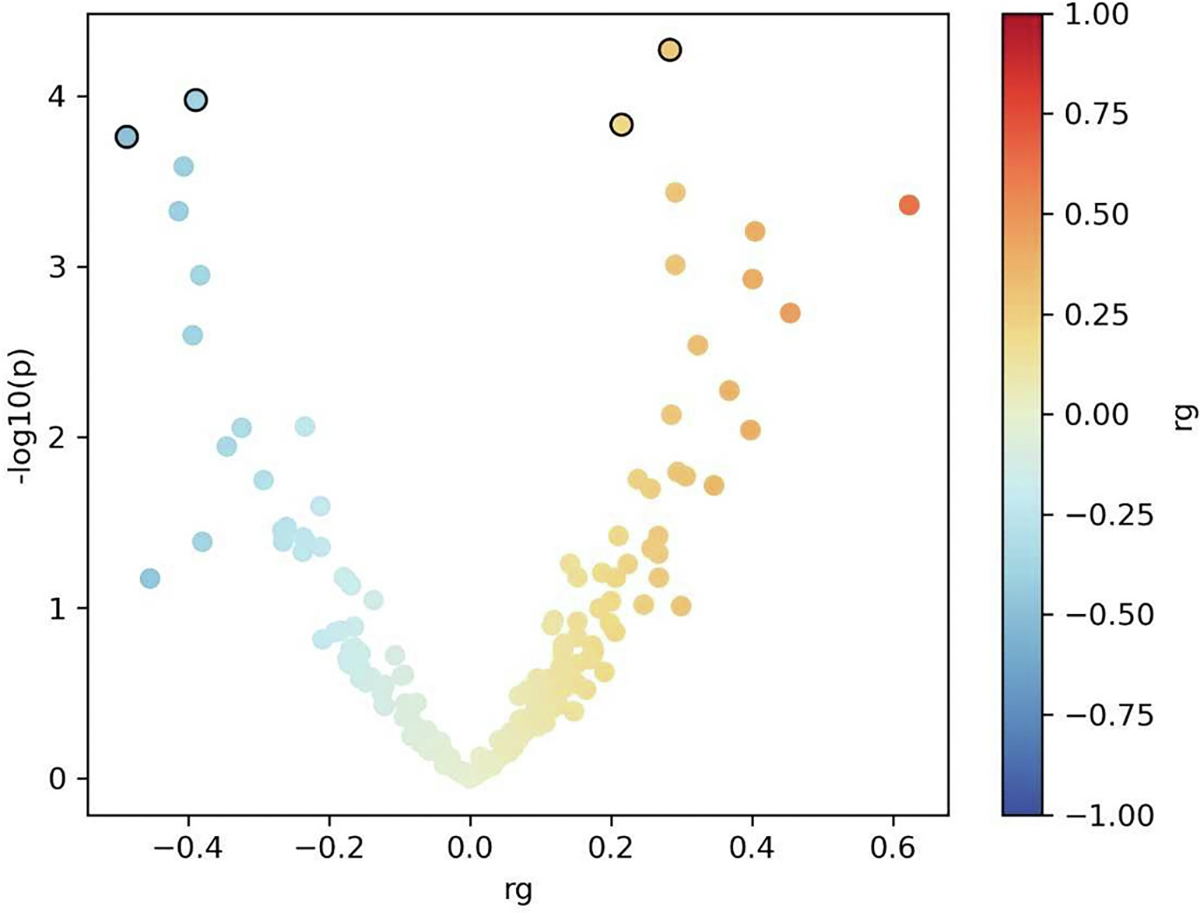
Volcano plot for local genetic correlation (rg) using LAVA for Alzheimer’s disease (AD) and multiple sclerosis (MS). 208 regions with significant h2 (p < 0.05/2495, where 2495 is the number of tested genomic regions) are shown (blue dots negative correlation, orange dots positive correlation), 4 regions have significant rg after Bonferroni correction (p < 0.05/208, outlined dots). (For interpretation of the references to colour in this figure legend, the reader is referred to the web version of this article.)

Regional genetic correlations between two traits may be masked when rg are assessed on a genome-wide level. To investigate if despite negligible genome-wide rg there are any genomic loci with pronounced genetic correlations, we estimated regional genetic correlations using LAVA (Werme et al., 2022). LAVA analysis revealed 208 genetic regions on different chromosomes spanning 253 Mb with significant h2 (*p* < 0.05/4595) in both AD and MS and a balanced mixture of positive (114) and negative (95) genetic correlations. Among 208 regions with significant h2, there were significant (p < 0.05/208) correlations at 4 loci: [chr 1:200134006–201,067,952, chr 5:87943483–89,584,466, chr 6:31250557–31,320,268, chr 16:11022543–11,916,631] (Table 4 Supplementary, Fig. 3).

### 3.3. ConjFDR analysis identify shared genomic loci between AD and MS

**Fig. 4.**
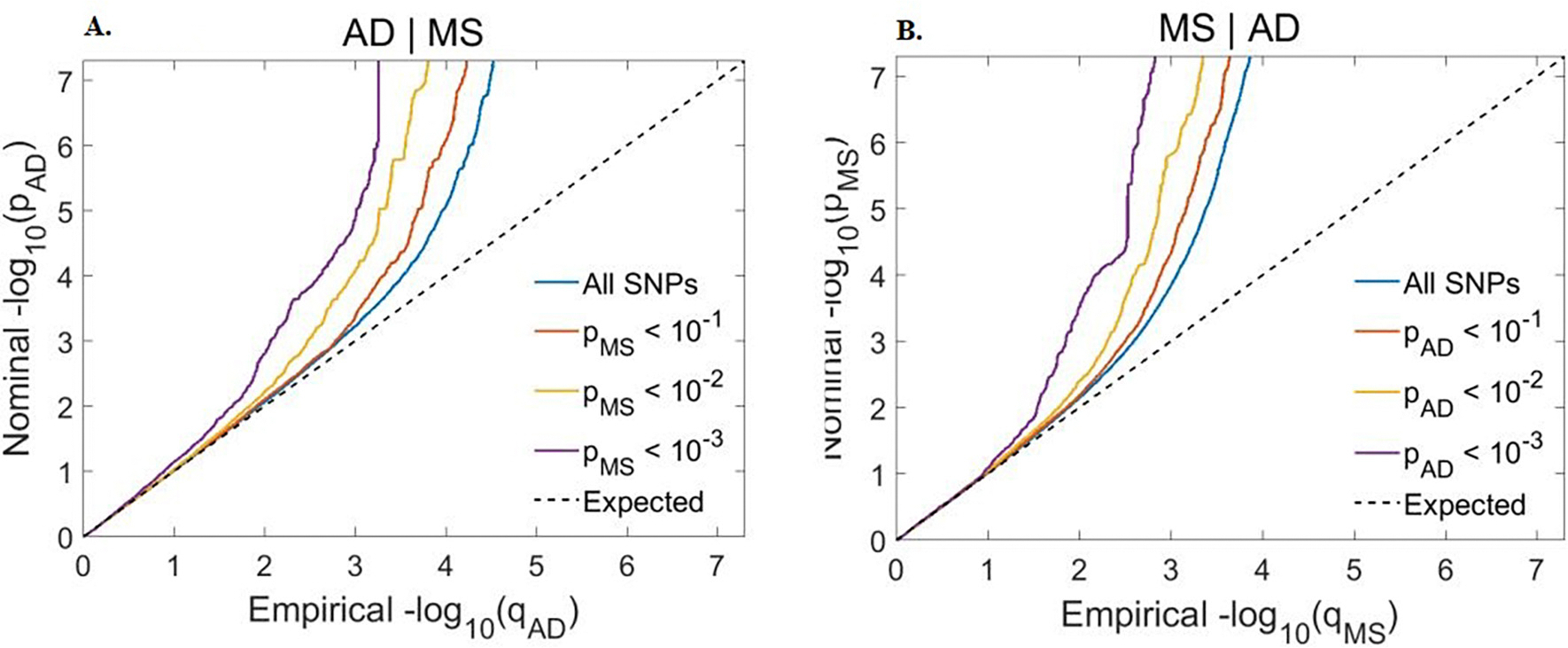
Conditional Q-Q plot shows the relation between expected (x axis) and observed (y axis) significance of variants in the primary phenotype when variants are stratified by their *p*-values in the conditional phenotype. A sequence of 4 nested strata is presented: all SNPs (blue), p_conditional_phenotype_ < 0.1 (red), p_conditional_phenotype_ < 0.01 (orange) and p_conditional_phenotype_ < 0.001 (purple). Dashed black line demonstrates expected behaviour under no association. Increasing degree of leftward deflection from the no-association line for strata of SNPs with higher significance in the conditional phenotype indicates polygenic overlap. A: Alzheimer’s disease (AD) conditioned on multiple sclerosis (MS). B: MS conditioned on AD. (For interpretation of the references to colour in this figure legend, the reader is referred to the web version of this article.)

Conditional Q-Q plots for AD and MS revealed strong enrichment in both directions (Fig. 4). Observed significant leftward shift for the group of SNPs with higher significance indicated genetic enrichment and possible shared genetic background between AD and MS. After the application of condFDR for both traits we showed comparable power to unconditional FDR analysis (Manhattan plot, Supplementary 1, Figs. 2 and 3).

**Fig. 5.**
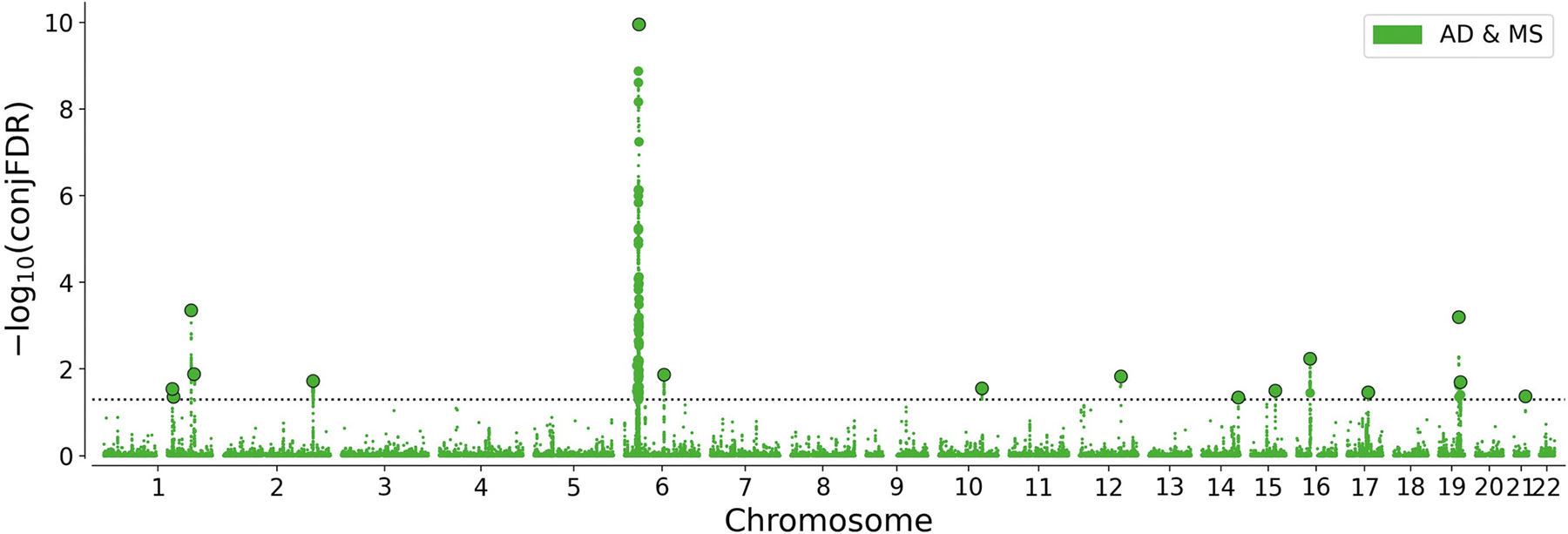
Chromosomal distribution of genetic loci jointly associated with Alzheimer’s disease (AD) and multiple sclerosis (MS). Manhattan plot shows the −log10 transformed conjunctional FDR values for AD and MS (green) for each SNP (*y*-axis) against chromosomal position (*x*-axis). The dotted line represents the conjFDR threshold for significant association <0.05. Lead variants at each locus are represented as bold dots with black borders. (For interpretation of the references to colour in this figure legend, the reader is referred to the web version of this article.)

ConjFDR analysis identified 16 loci jointly associated with AD and MS (see Manhattan plot at Fig. 5, Table 1). Six of them were novel for AD and three for MS according to GWAS catalog and novelty checking protocol. Furthermore, 50% of lead SNPs (8/16) had the same effect direction on MS and AD. These results are consistent with the observation of data low global genetic correlation between two diseases because SNPs have mixed directions of effects, which was also supported by the LAVA results.

To replicate our genetic findings, we used summary statistics from an independent Afro-American cohort with AD. One locus was nominally significant in the independent cohort but did not survive the multiple testing correction (rs35866622, p_replication = 0.0037). Other loci identified in our conjFDR analysis were non-significant in the independent Afro-American cohort at both nominal and multiple testing correction levels.

### 3.4. Functional annotation of identified loci

Positional mapping with FUMA showed that most of the identified lead variants were intronic (68.8%, 11/16) (Table 1, Fig. 6, Supplementary, A). One of the lead SNPs (rs35866622 in *MAMSTR* gene) has CADD score above 12.37, suggesting high deleteriousness. This lead SNP was nominally significant in the Afro-American population. The Locus zoom plot for this variant and circos plot for chromosome 19 are presented at Figs. 4 and 5, A (Supplementary).

According to Regulome Database classification, the rs10400902 (*CTSH* gene) variant can affect binding. Two other SNPs (rs 1,846,190 and rs10806425) in *HLA-DQA1* and *BACH2* have classification 3a and are less likely to affect binding.

Then we performed analysis using Open Targets platform to aggregate evidence linking variants to genes and revealed that in 8 cases other genes than in FUMA were mapped according to Open Targets algorithm (presented at Table 1 as the second gene in column “Gene” and in Table 2 in Supplementary for results from weighted models and pQTL, eQTL, sQTL). We used the gene-to-function analysis in FUMA for all genes highlighted by either FUMA or Open Targets (24 genes) to understand which pathways and tissues might be involved. Five identified genes were highly expressed in brain tissue – *ALDOA*, *COPA*, however these genes also revealed high expression rates in other tissues. *BACH2*, *FAM117B* genes had elevated expression during early prenatal period and for *ETS2* in adulthood (Supplementary Fig. 5., B). Evaluated genes were up-regulated in the spleen using GTEx 30 general tissue types database (Supplementary Fig. 6, B). Additionally, we performed MAGMA analysis for MS and AD summary statistics and revealed two common tissues (spleen and whole blood) using GTEx 54 general tissue types database (Supplementary Fig. 7, for spleen *p* = 2.1949e-05 and *p* = 1.0743e-17, for whole blood *p* = 0.00010933 and *p* = 7.7985e-13 for AD and MS, respectively).

Using pathway analysis, only molecular signatures database revealed several significant changes: according to positional gene sets (MsigDB c1) which reflect the gene architecture and chromosomal changes there are structural changes at chromosome 1 (Chr1q23) for genes *FCRL2*, *FCRL1*, *COPA*, *NHLH1* (see Supplementary Fig. 6.C).

According to Immunologic signatures (MsigDB c7) a lot of immunological pathways are implicated (Supplementary Fig. 8 and 9) that confirm shared immunology between disorders (Godec et al., 2016).

## 4. Discussion

Here we applied state-of-the-art statistical tools to improve our knowledge of the genetic underpinnings of the relationship between AD and MS. According to our results, the polygenicity of AD and MS was similar, but less than for other psychiatric disorders (Smeland et al., 2020; Hindley et al., 2022). Substantial genetic overlap between AD and MS was revealed using MiXeR, estimated to 400 trait-influencing variants, using an AD-specific setup of the MiXeR previously published by Holland D (Holland et al., 2020). In this setup, chromosome 19 is analysed separately, to mitigate potential violations of the MiXeR model assumptions due to the extreme significance of the *APOE* region in AD. As demonstrated previously, this setup improves model fitness and provides higher sensitivity to polygenic components.

A total of 16 shared genetic loci were revealed by the cFDR approach using current GWAS data. Annotated genes in shared loci were enriched in molecular signalling pathways involved in inflammation and the structural organisation of neurons. Taken together, these findings support a shared genetic architecture between AD and MS, and implicate novel molecular pathways. Despite the observed substantial genetic overlap, the genetic correlation between AD and MS was negligible and insignificant, suggesting a mixed direction of effects in the shared loci. This is supported by the LAVA analysis revealing a balanced mixture of concordant and discordant shared loci scattered throughout the genome. These findings indicate that common variants that influence the genetic risk for diverse neurological phenotypes may have risk-enhancing and risk-reducing effects on different disorders or be involved in the same processes with different effect directions on disease. We hypothesise that immunological pathways are influenced in different ways: in MS immunological activation can lead to relapse (Wei et al., 2021), while in AD it can be connected to protective health-related immune perturbation and maintain proper metabolic clearance (Piehl et al., 2022; Cisbani and Rivest, 2021). Additionally, it can be linked to the idea that microglia can play both neuroprotective and deleterious roles in neurological disease pathogenesis: Aβ and damaged myelin can prompt a transcriptional shift in microglia to promote the upregulation of microglial phagocytic machinery to clear the pathology (Ennerfelt and Lukens, 2023). Immunological involvement might confirm the effectiveness of humanised monoclonal antibodies in AD therapies, such as lecanemab (https://www.fda.gov/news-events/press-announcements/fda-grants-accelerated-approval-alzheimers-disease-treatment). Moreover, unearthing the mechanism by which the immunological system can modify pathology in neurodegenerative diseases can be important for future directions of drug development.

The immune system’s involvement in the pathogenesis of late-onset AD was suggested by the recent AD GWASs (Wightman et al., 2021; Jansen et al., 2019; Bellenguez et al., 2022). In our study FUMA gene to function analysis revealed an essential component of immunological function according to Molecular Signatures Database, and FUMA tissue specificity analysis for shared loci revealed the involvement of spleen in both disorders. These results were confirmed by MAGMA which displayed significant enrichment in spleen and blood tissue for both conditions supporting previous studies (Jansen et al., 2019; Wightman et al., 2021). Furthermore, it pointed to the immune system’s involvement in both diseases. Additionally, the present study showed the involvement of shared immune-related gene loci between AD and MS and identified several novel loci. *FCRL1* and *FCRL2* genes were previously described as involved in AD pathophysiology (Cohen et al., 2020) and have an essential role in immunological pathways. These genes encode a member of the immunoglobulin receptor superfamily and are one of several Fc receptor-like glycoproteins clustered on the long arm of chromosome 1. This locus on chromosome 1 was previously described in MS (International Multiple Sclerosis Genetics Consortium (IMSGC) et al., 2013) but it is novel for AD.

*COPA* was reported to be an essential gene in developing of systemic immune disorders such as psoriasis, lung immune disorders, and others (Watkin et al., 2015), and it can be linked to immune pathology in MS and AD. Other genes potentially involved in immune system pathogenesis include *BACH2*, which is associated with NF-kappa b signalling (Boche and Gordon, 2022), *BLNK*, which is a B-cell linker protein and is associated with B-cell signalling (Turkoglu et al., 2021), and *CR1*, a complement C3b/C4b receptor 1 involved in different complement associated processes. Decreased expression of CR1 protein was shown to occur in systemic lupus erythematosus, sarcoidosis, and AD (Crehan et al., 2012). *PVR,* or *CD155*, belongs to a large family of immunoglobulin (Ig)-like molecules called nectins and nectin-like proteins, which mediate cell-cell adhesion, cell migration, and cell polarisation by interacting with other nectins, and has also been established as an immunomodulatory receptor, which is able to activate and inhibit natural killer cells (Lupo and Matosevic, 2020).

Additionally, HLA involvement links to the importance of inflammatory pathways in the pathogenesis of both diseases as previously described (Lindbohm et al., 2022). Using Cytoscape and the STRING protein database (Szklarczyk et al., 2017), we revealed interactions between HLA, CTSH and BATCH2 proteins which are strongly linked to the immune system and protein metabolism pathways (Fig. 9., Supplementary). In both diseases, it was linked with the same effect direction in cFDR and LAVA. However, it should be treated with caution due to intricate LD structure in this region. The knowledge of the new immune genes potentially involved in AD pathology can be linked to new drug development. After the discovery of TREM2 mutations the study of a new target drug has been started (A Phase 2 Study to Evaluate Efficacy and Safety of AL002 in Participants With Early Alzheimer’s Disease - Full Text View - ClinicalTrials.gov, NCT04592874). Moreover, many monoclonal antibodies to different targets entered phase 1 and 2 of clinical trials during the last years (Cummingd et al., 2022). Once more confirmatory studies on the role of neuroinflammation in AD are conducted, the development of new AD medications in this area could accelerate (Olloquequi et al., 2022).

In addition to the immune system, other processes are shared between the two disorders. *MAPK3* is involved in the phosphorylation of tau proteins microtubule-associated protein 2 and 4, which link to microtubules system and neurodegeneration (Lund et al., 2014). *PLXNC1* encodes a member of the plexin family, which is involved in the regulation of axon guidance, cell motility and migration, and the immune response. *CTSH* encoded a cathepsin H protein which can be involved in lysosomal pathology and neurodegeneration (Drobny et al., 2022). Functional annotation highlighted a SNP on chromosome 19 with a highly deleterious CADD score, which contains *MAMSTR* and is replicated in an independent study. The mutation has different directions in AD and MS, and the involvement of this SNP was described previously.

A gap between the number of shared trait-influencing variants predicted by MiXeR and the number of shared loci identified by the cFDR approach can be attributed to undiscoverable “missing” heritability due to current insufficient GWAS power (Wightman et al., 2021), rare and structural variants (Matthews and Turkheimer, 2022; Andrews et al., 2023). Furthermore, a substantial increase in current sample sizes is required for both disorders to detect most of the common risk variation at the genome-wide significance level. The heritability for AD was lower than for MS. The small difference between AD heritability estimates obtained with MiXeR and LDSC methods might be attributed to the different mathematical models underlying these methods (Frei et al., 2019; Bulik-Sullivan et al., 2015). LDSC is based on the infinitesimal model which assume that each variant has an effect on the phenotype (Bulik-Sullivan et al., 2015), which might be suboptimal for late-onset neurological disorders with complex genetic architecture (Baker et al., 2023). On the other hand, MiXeR assumes that only a fraction of variants influences the phenotype while remaining genomic variants have zero effect.

This study has some limitations. Due to the inclusion of the most recent GWAS data for both AD and MS in our discovery analysis, our replication analysis was limited to a small AD GWAS based on a sample with different ancestry, which can explain the low replication level. Although genetics plays a substantial role in the etiology of AD and MS, and there is genetic overlap between the disorders, the part of environmental influences should not be omitted as well as differences in neuropathological processes due to gene-environment interplay, epistatic effects and other pathological pathways involved in the pathogenesis of AD and MS.

In summary, the main findings of our study were comparable polygenicities for AD and MS with 20% of trait-influencing variants being shared between the disease despite negligible global genetic correlation. ConjFDR analysis identified 16 shared genetic loci between AD and MS, with 8 having concordant direction of effects. Annotated genes in shared loci were enriched in molecular signalling pathways involved in inflammation and the structural organisation of neurons. These findings provide molecular genetic insights into the immune mechanisms involved in AD.

## Supplementary Material

Supplementary_figures

1-s2.0-S0969996123001894-mmc2

## Figures and Tables

**Table 1:** Genomic loci are jointly associated with Alzheimer’s disease (AD) and multiple sclerosis (MS) at conjunctional FDR (conjFDR) < 0.05.

Locus	Lead SNP	Chr	Lead BP	A1/A2	Nearest Gene by FUMA, Top gene by open targets*	distance	Functional category	CADD	RDB	P-val. AD	Z score AD	P-val. MS	OR MS	Z score MS	conjFDR	Novel_in_AD	Novel in MS	p in Kunkle et al

1	rs6675393	1	157,734,907	C/T	FCRL2, *FCRL1*	0	intronic	1.242	7	1.46E-04	3.798	2.48E-06	1.0821	4.7102	2.86E-02	Yes	No	0.078
2	rs2147472	1	160,341,876	G/A	NHLH1, *COPA*	0	UTR3	1.638	5	1.86E-04	3.737	4.60E-04	1.061	3.5008	4.37E-02	No	No	0.1038
3	rs12122809	1	201,014,966	C/T	CACNA1S	0	intronic	0.499	7	9.46E-07	−4.903	3.38E-09	1.114	5.9122	4.31E-04	Yes	No	0.3816
4	rs6661489	1	207,698,044	C/T	CR1	0	intronic	1.175	7	4.47E-14	−7.546	8.10E-05	0.9101	−3.9414	1.28E-02	No	Yes	no
5	rs72932709	2	203,639,501	A/G	AC098831.4, *FAM117B*	417	upstream	0.611	5	7.84E-05	3.949	1.36E-04	1.0991	3.8146	1.86E-02	No	No	0.48
6	rs1846190	6	32,583,813	G/A	HLA-DQA1, *HLA-DQA2*	12,142	intergenic	11.31	3a	2.81E-13	7.303	2.23E-99	1.9759	21.16	1.10E-10	No	No	no
7	rs10806425	6	90,926,612	A/C	BACH2	0	intronic	2.974	3a	4.94E-05	4.059	4.49E-06	1.0791	4.5874	1.33E-02	Yes	No	0.3872
8	rs1870171	10	98,019,113	G/A	BLNK	0	intronic	4.612	5	1.07E-04	3.875	2.42E-04	0.8954	3.67	2.76E-02	Yes	Yes	0.77
9	rs2242498	12	94,649,595	C/T	PLXNC1	0	intronic	0.656	5	3.49E-05	−4.139	9.80E-05	1.0663	3.8955	1.46E-02	Yes	No	0.941
10	rs17617994	14	103,959,289	G/T	MARK3	0	intronic	0.094	7	2.84E-04	3.629	3.74E-05	0.9073	−4.1233	4.47E-02	Yes	No	0.87
11	rs10400902	15	79,231,616	G/A	CTSH	0	intronic	5.081	1d	1.68E-04	3.763	2.54E-04	1.0665	3.6585	3.14E-02	No	No	0.421
12	rs9939774	16	30,068,354	C/T	ALDOA, *INO80E*	0	intronic	12.11	6	1.02E-05	4.414	2.64E-05	0.9319	−4.2029	5.66E-03	No	No	0.3138
13	rs2119931	17	47,429,834	G/A	ZNF652, *PHOSPHO1*	0	intronic	7.636	6	1.89E-04	3.733	1.42E-04	0.9354	−3.8052	3.40E-02	No	Yes	0.33
14	rs7260482	19	45,143,942	A/C	CTB-171A8.1, *PVR*	0	ncRNA_intronic	1.443	4	6.92E-18	8.616	1.84E-06	0.9152	−4.7704	6.20E-04	No	No	0.4268
15	rs35866622	19	49,218,060	C/T	MAMSTR	0	intronic	13.01	4	2.29E-05	−4.234	1.51E-04	1.0671	3.7897	1.98E-02	No	No	0.003679
16	rs2094871	21	40,460,859	G/A	RPL23AP12, *ETS2*	38,634	intergenic	2.31	4	2.07E-04	−3.71	4.40E-04	1.0645	3.5146	4.21E-02	No	No	0.7006

For each identified locus the table presents the rs number of the lead SNP, its chromosomal position and alleles, the nearest gene and its functional category, and the number of base pairs (distance) between the lead SNP to the gene according to FUMA, as well as p-values and effect sizes (odds ratios for MS, z-scores for AD) from the original summary statistics on AD and MS. The effect sizes are given with reference to allele 1 (A1). CADD score was used for predicting the deleteriousness of variants. Regulome database (RDB) score was used for functional annotation. Columns “Novel_in_” means novelty for SNP in the disease. Column p in “Kunkle_et_al” presented *p* value in replication dataset. Significant value marked in bold.

* At column 6 the first gene positionally mapped by FUMA, the second gene marked in *cursive* is a top gene defined by Open Targets. If one gene is marked in bold it means that FUMA and Open Targets predict the same gene.

## Data Availability

GWAS data without IGAP and 23andme from PGC-ALZ were downloaded: https://www.med.unc.edu/pgc/download-results/ and additionally modified by request. GWAS data for IMSGC can be obtained after request to IMSGC data access committee: https://imsgc.net. Publicly available summary statistics for Afro-American with *p*-value were received from NG00100 - Alzheimer’s disease in African Americans(NIAGADS). Statistical analyses were performed in MATLAB and Python, using existing tools available on GitHub, including conditional/conjunctional false discovery rate (https://github.com/precimed/pleiofdr).

## Abbreviations:

AD: Alzheimer’s disease
Aβ: amyloid-beta
AIC: Akaike information criterion
APOE: apolipoprotein E
CADD: Combined Annotation Dependent Depletion
cFDR: conditional/conjunctional false discovery rate framework
cond/conjFDR: conditional/conjunctional false discovery rate
eQTL: expression quantitative trait locus
GWAS: genome-wide association studies
h2: SNP heritability
LAVA: Local Analysis of [co]Variant Association
LD: linkage disequilibrium
LDSC: LD score regression
MAPT: microtubule-associated protein tau gene
MHC: major histocompatibility complex
MiXeR: causal mixture model
MS: Multiple sclerosis
PGC: Psychiatric Genomic Consortium
pQTL: protein quantitative trait locus
