# Supplementary material for: Shared genetic loci between Alzheimer’s disease and multiple sclerosis: Crossroads between neurodegeneration and immune system
Source: Neurobiol Dis. Author manuscript; Available in PMC 2025 Mar 6. (PMC11884797; doi:10.1016/j.nbd.2023.106174)
Supplement: Supplementary_figures [file NIHMS2047170-supplement-Supplementary_figures.pptx]

## Slide 1
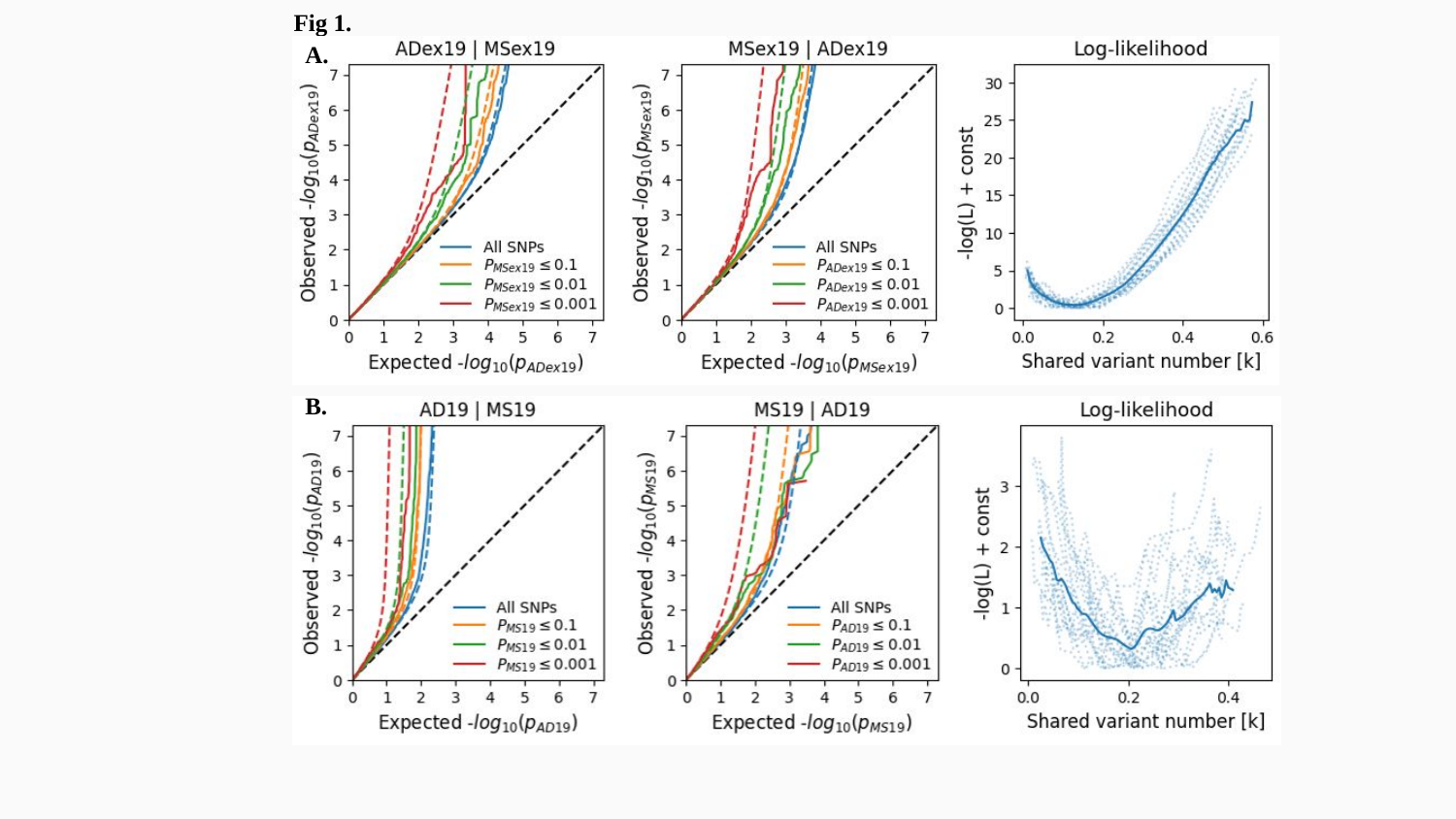

Fig 1.
A.
B.

## Slide 2
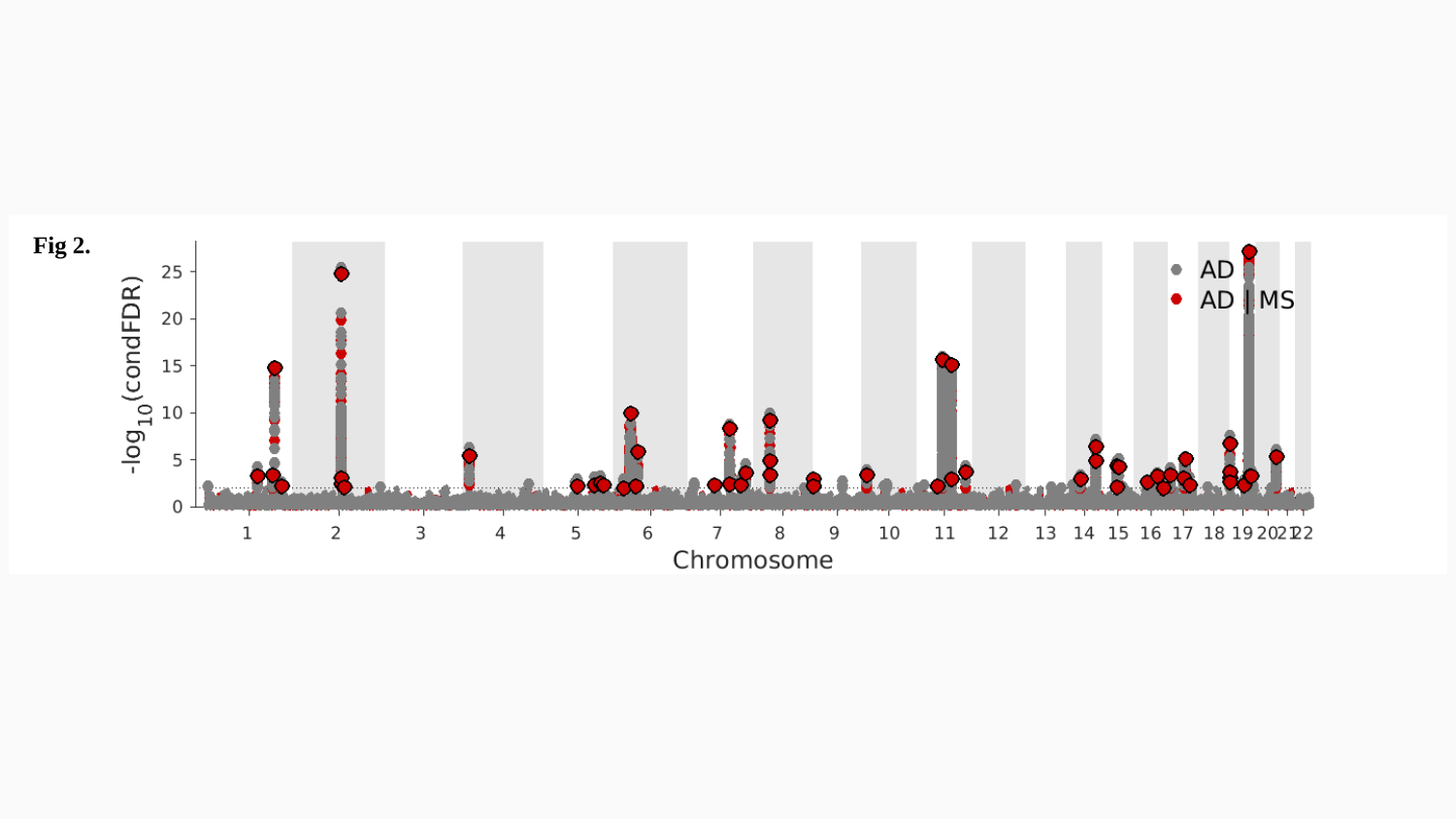

Fig 2.

## Slide 3
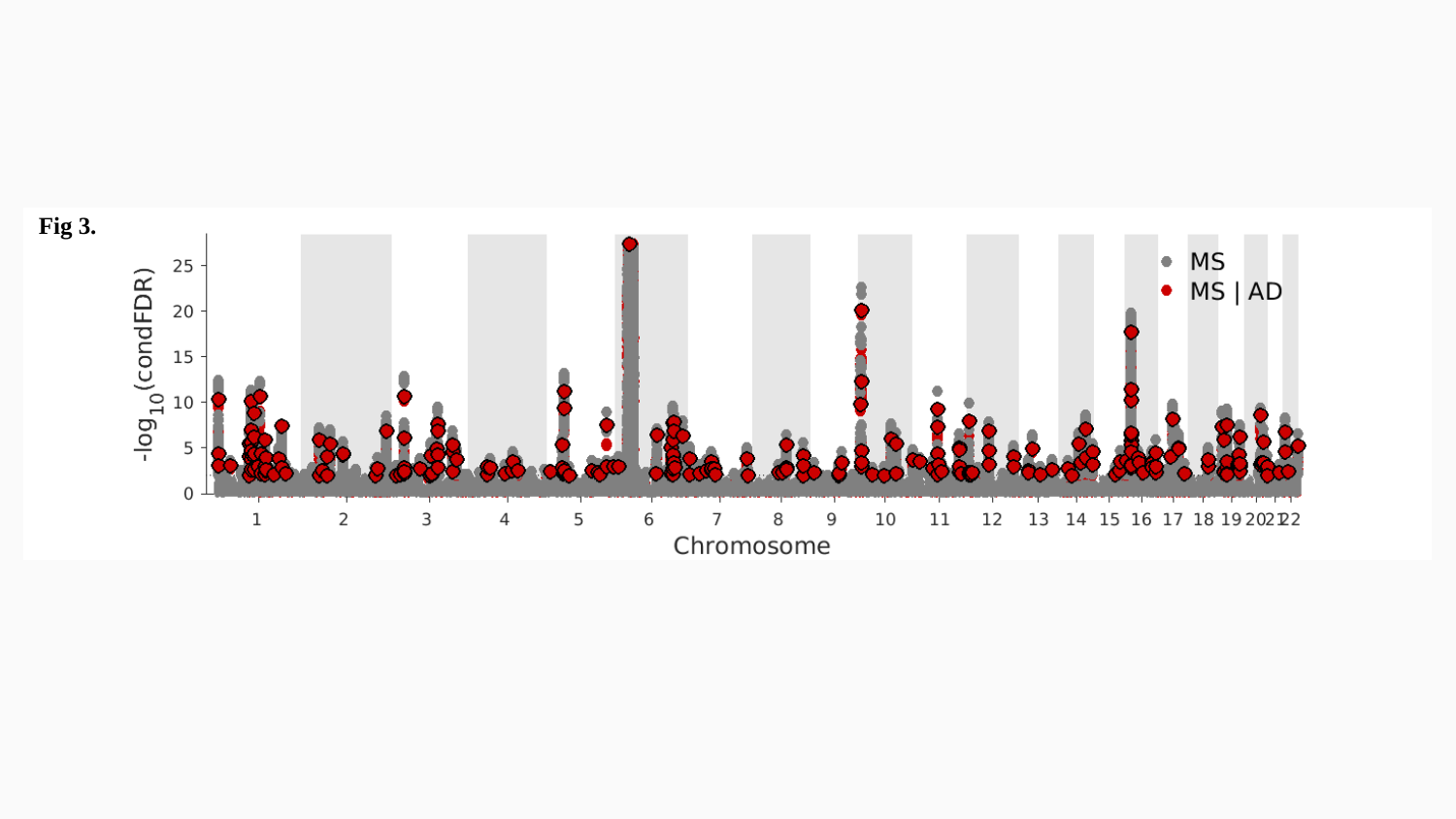

Fig 3.

## Slide 4
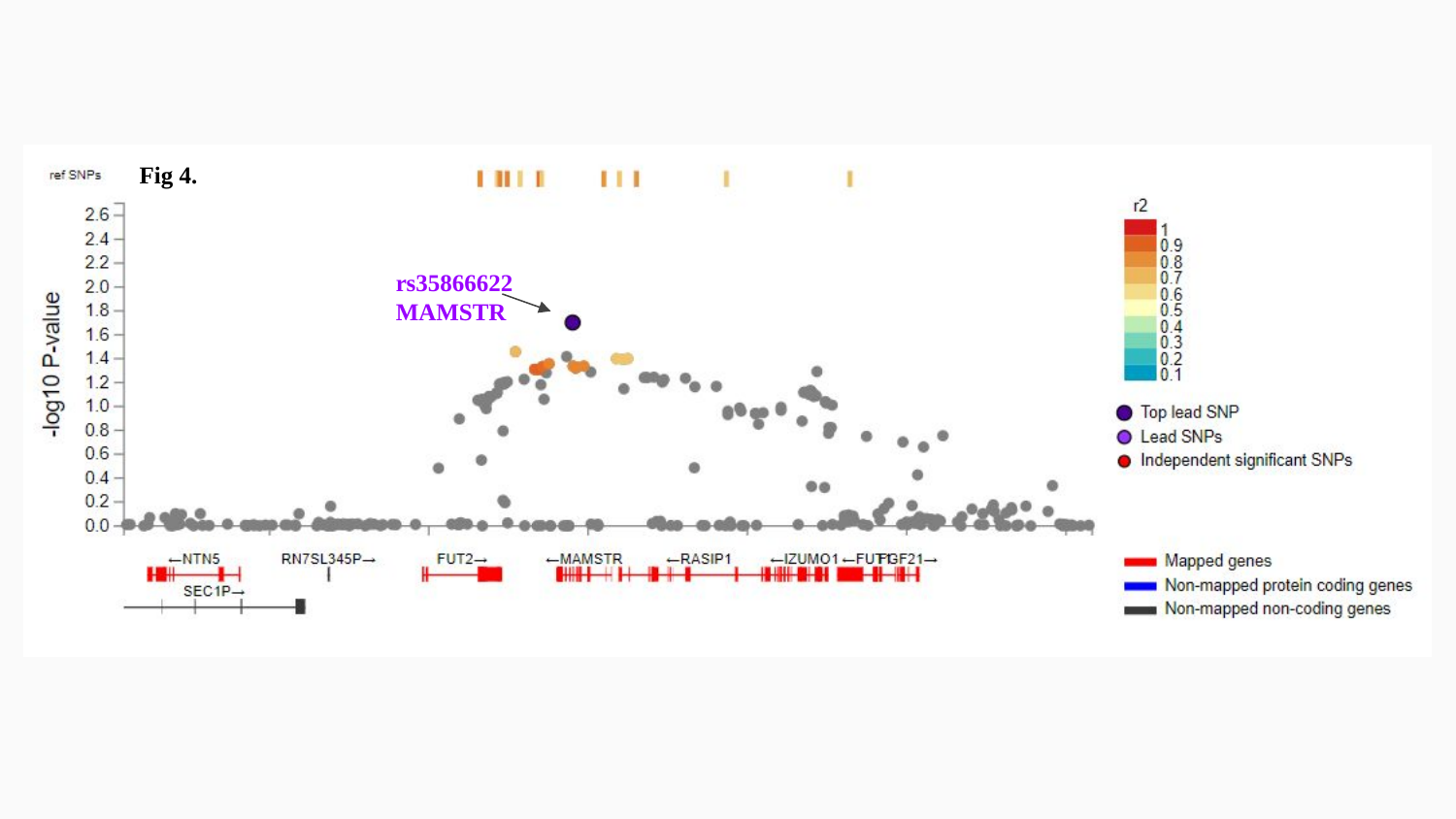

Fig 4.
rs35866622
MAMSTR

## Slide 5
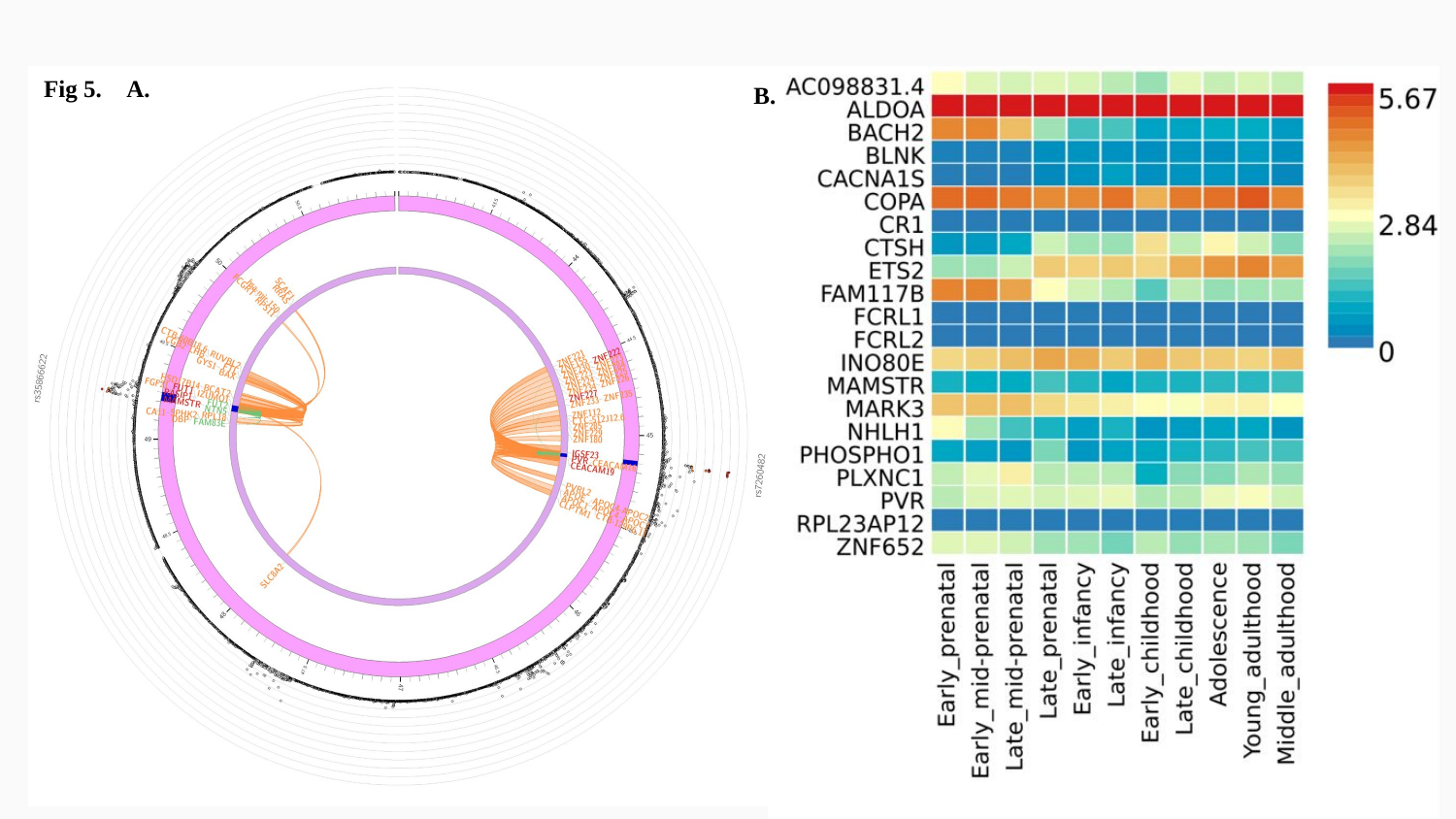

A.
Fig 5.
B.

## Slide 6
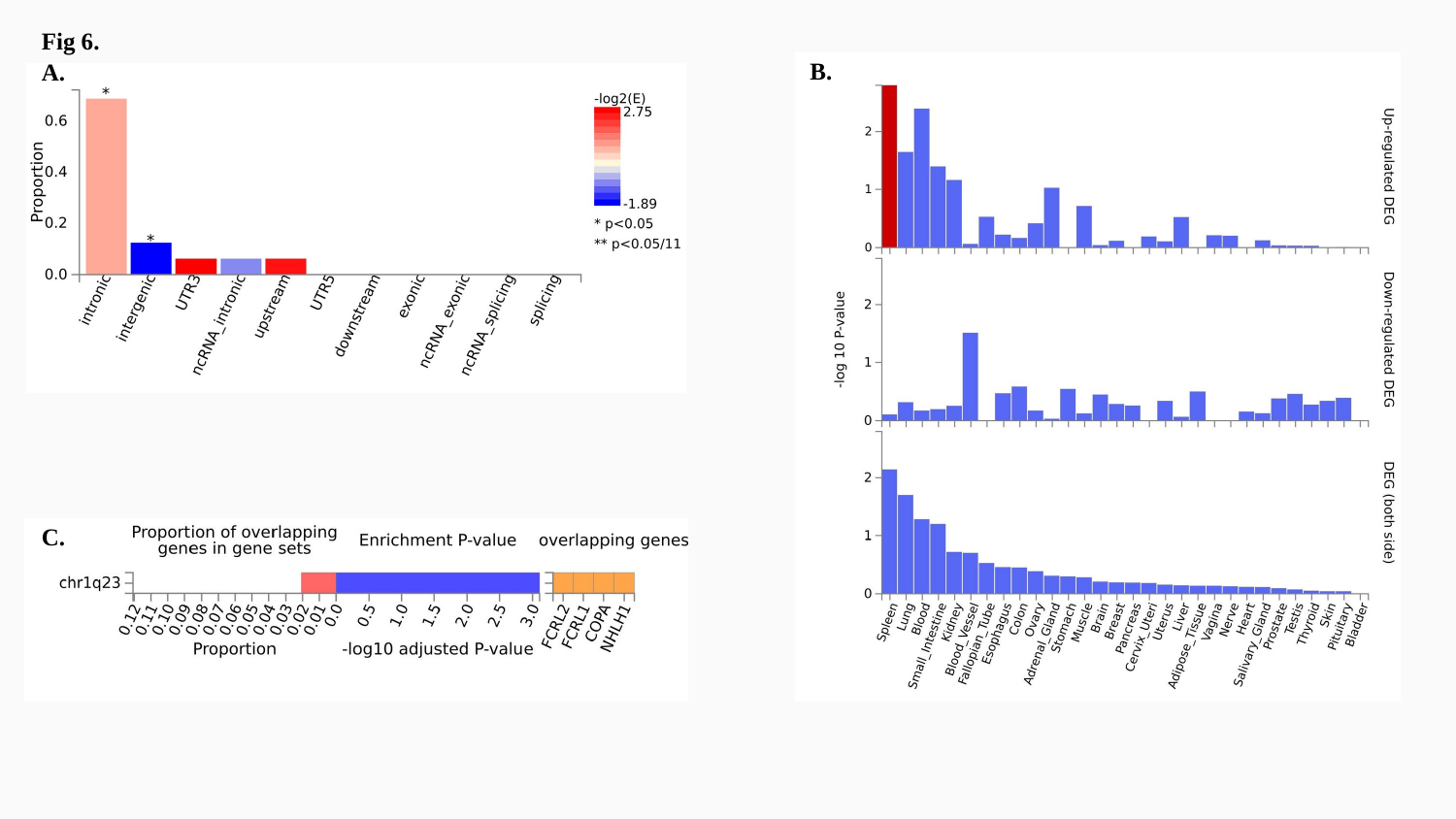

Fig 6.
B.
A.
C.

## Slide 7
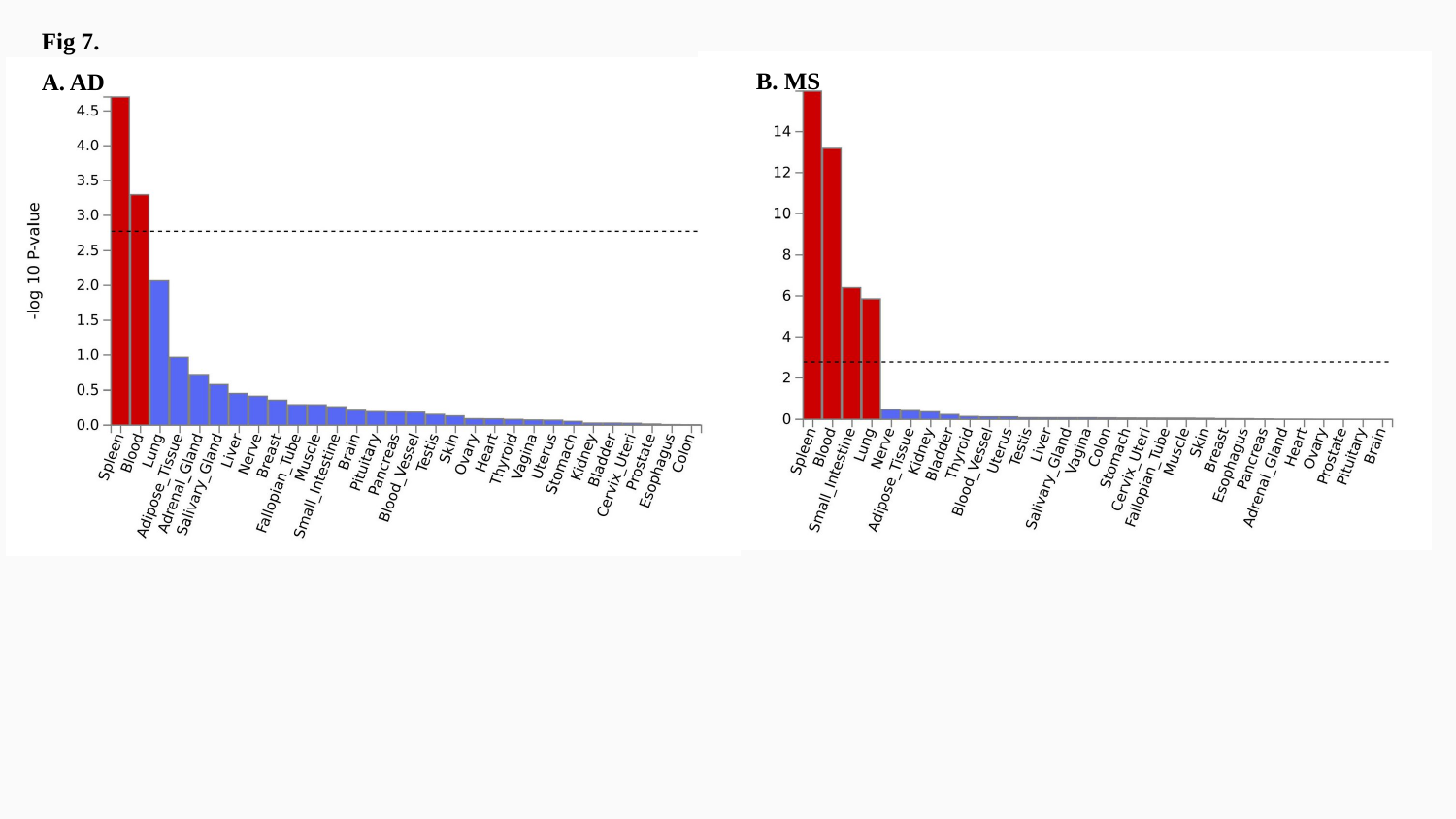

Fig 7.
B. MS
A. AD

## Slide 8
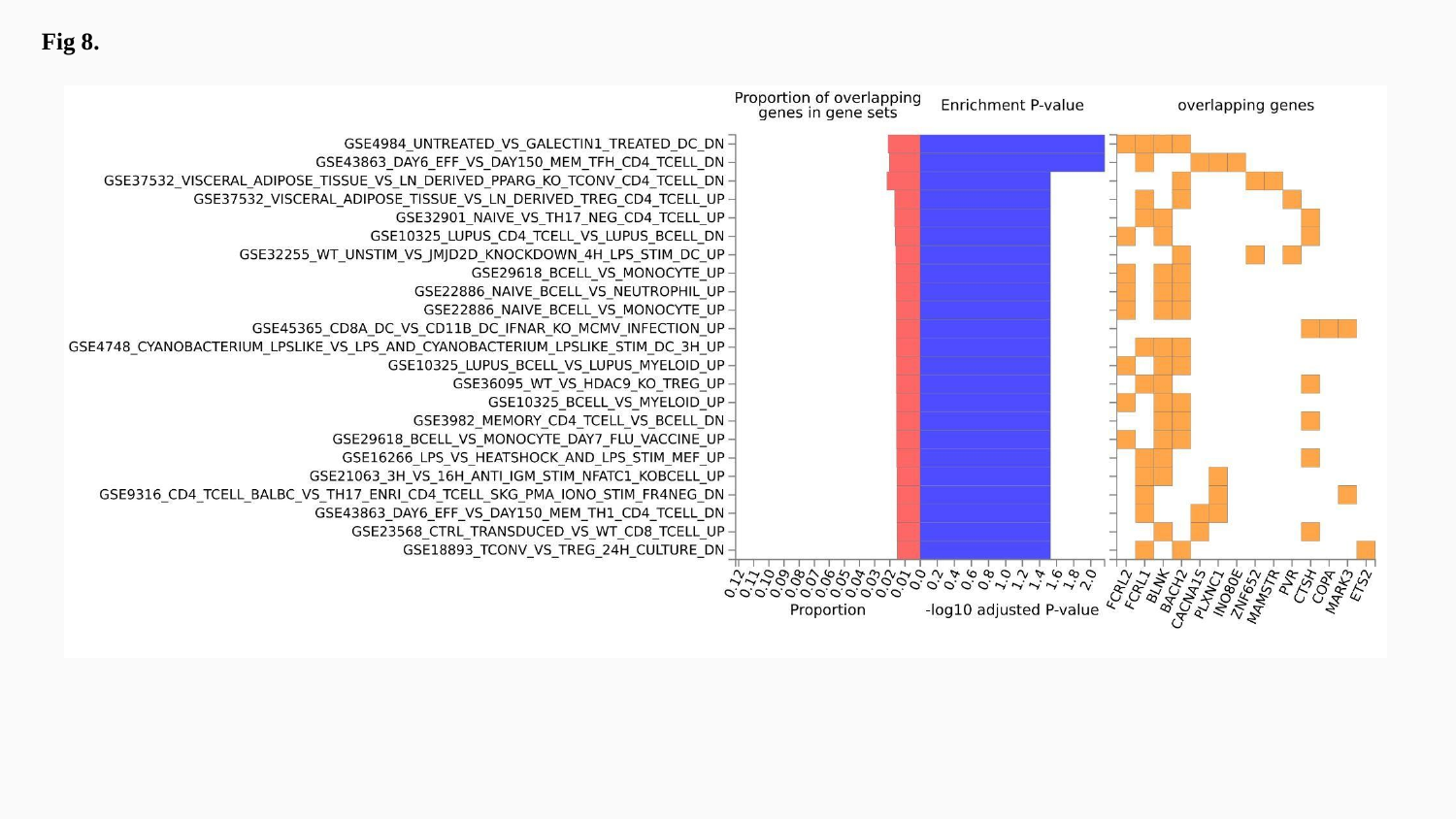

Fig 8.

## Slide 9
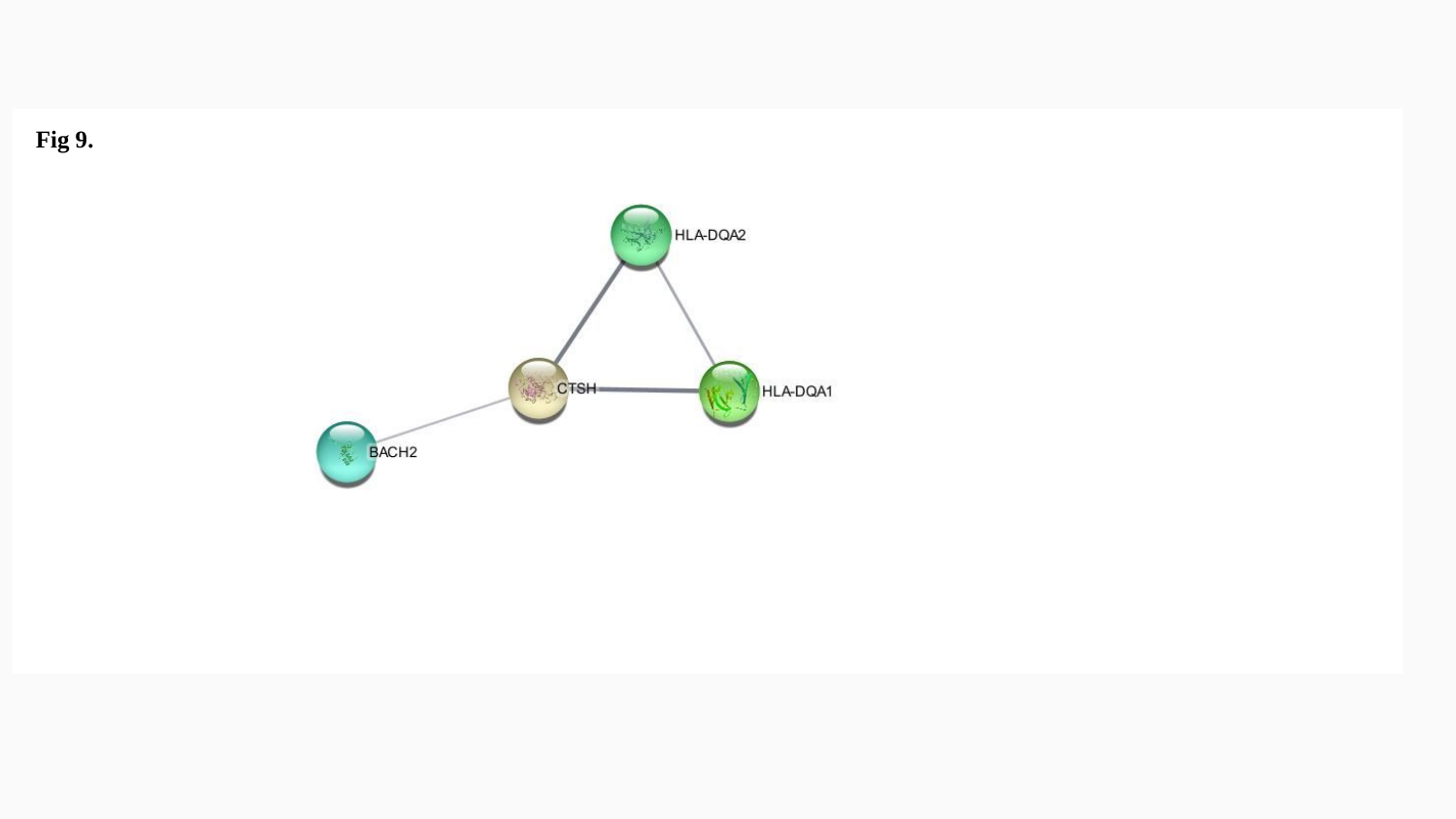

Fig 9.
